# Identification of subgroups along the glycolysis-cholesterol synthesis axis and the development of an associated prognostic risk model

**DOI:** 10.1186/s40246-021-00350-3

**Published:** 2021-08-12

**Authors:** Enchong Zhang, Yijing Chen, Shurui Bao, Xueying Hou, Jing Hu, Oscar Yong Nan Mu, Yongsheng Song, Liping Shan

**Affiliations:** 1 Department of Urology, Shengjing Hospital of China Medical University, Shenyang, 110004 Liaoning China; 2 Department of Otolaryngology Head and Neck Surgery, Shengjing Hospital of China Medical University, Shenyang, Liaoning China; 3 School of Postgraduate, China Medical University, Shenyang, Liaoning China; 4 Department of Breast Surgery, Shengjing Hospital of China Medical University, Shenyang, China; 5 Englewood Hospital and Medical Center, Englewood, NJ USA

**Keywords:** Melanoma, Predictive model, Metabolic, Machine learning

## Abstract

**Background:**

Skin cutaneous melanoma (SKCM) is one of the most highly prevalent and complicated malignancies. Glycolysis and cholesterogenesis pathways both play important roles in cancer metabolic adaptations. The main aims of this study are to subtype SKCM based on glycolytic and cholesterogenic genes and to build a clinical outcome predictive algorithm based on the subtypes.

**Methods:**

A dataset with 471 SKCM specimens was downloaded from The Cancer Genome Atlas (TCGA) database. We extracted and clustered genes from the Molecular Signatures Database v7.2 and acquired co-expressed glycolytic and cholesterogenic genes. We then subtyped the SKCM samples and validated the efficacy of subtypes with respect to simple nucleotide variations (SNVs), copy number variation (CNV), patients’ survival statuses, tumor microenvironment, and proliferation scores. We also constructed a risk score model based on metabolic subclassification and verified the model using validating datasets. Finally, we explored potential drugs for high-risk SKCM patients.

**Results:**

SKCM patients were divided into four subtype groups: glycolytic, cholesterogenic, mixed, and quiescent subgroups. The glycolytic subtype had the worst prognosis and *MGAM* SNV extent. Compared with the cholesterogenic subgroup, the glycolytic subgroup had higher rates of *DDR2* and *TPR* CNV and higher proliferation scores and MK167 expression levels, but a lower tumor purity proportion. We constructed a forty-four-gene predictive signature and identified MST-321, SB-743921, Neuronal Differentiation Inducer III, romidepsin, vindesine, and YM-155 as high-sensitive drugs for high-risk SKCM patients.

**Conclusions:**

Subtyping SKCM patients via glycolytic and cholesterogenic genes was effective, and patients in the glycolytic-gene enriched group were found to have the worst outcome. A robust prognostic algorithm was developed to enhance clinical decisions in relation to drug administration.

## Background

Skin cutaneous melanoma (SKCM) is a highly aggressive malignancy, and the mortality rate increases dramatically following metastasis [1]. In its early stage, melanoma can be successfully treated with surgery; however, once it has metastasized it needs to be treated with drugs [2]. Adjuvant systemic therapy is widely used in melanoma patients, especially those with stage III/IV. Immune checkpoint inhibitors and BRAF-targeted therapies have shown efficacy in curbing metastatic melanoma [3], but they often fail in many SKCM patients due to drug insensitivity and resistance, which is attributed to the heterogeneity of melanoma [4].

Building molecular subtypes and a risk model algorithm for SKCM could be an effective solution to determining clinical pathways. In this respect, the Cancer Genome Atlas (TCGA) group established a framework for classifying genomes into four subtypes: mutant BRAF, mutant RAS, mutant NF1, and Triple-WT (wild-type) [5]. Another study identified two immune subtypes that have opposite immune states and developed a prognostic five-immune-associated gene signature [6]. In addition, a two-gene immune-related signature consisting of CCL8 and DEFB1 was constructed in 2020 [7], and the six genes (SH2D3A, TMEM201, LZTS1, CREG1, NIPA1 and HIST1H4E) model developed by another team are projected to play a vital role in the prognosis of uveal melanoma [8]. However, studies focusing on glycolysis-cholesterogenesis-related subtypes and associated prognostic models in melanoma are currently lacking.

Metabolic activity is pivotal to the developmental progress of tumors, and it contributes to tumor plasticity. The characteristics of tumor heterogeneity are reflected in cellular and metabolic aspects, including the differential tumor microenvironment (TME) and variable biological pathways [9, 10]. Altered metabolic activities influence tumor progress, reflect the associated prognosis, and influence the drug therapeutic effect [11]. Evidence has shown that glycolysis contributes to the disease progress of melanoma and that restricting glycolytic activity acts as a therapy, preserves immune cell function, and improves the immune checkpoint blockage effects [12]. The research of Andreas Koc et al. showed that the expression level of Cyclin D1, which acts as an outcome biomarker in melanoma and indicates its proliferative and invasive extents, is associated with glucose transporter isoform 1 (GLUT1), glycolysis-related genes hexokinase 1 (HK1), lactate dehydrogenase A (LDH-A), and monocarboxylate transporters 1 (MCT1) [13]. Many malignancies, such as lung, gastrointestinal, and pancreatic cancer, harbor KRAS and defunctional TP53 oncogenes, both of which induce glycolytic pathways in malignancies [14–17]. Unlike healthy tissues, most cancer cells mainly produce energy through glycolysis at a high rate, and at the same time metabolite pyruvate is converted to lactic acid followed by fermentation in cytosol. This process is known as the Warburg effect; it is a hallmark of tumor evolution and it impacts drug efficacy [18].

The Mitochondrial pyruvate complex (MPC) can counteract the influence of glycolytic activity by transporting pyruvate to mitochondrion, and loss-of-function MPC is related to fast cancer cell growth and a poor outcome [19]. In the metabolic process, pyruvate, which is an intermediate molecule of the tricarboxylic acid cycle (TCA cycle), provides precursor citrate for further adipogenesis, including synthetic cholesterol and free fatty acids [20]. Evidence has shown that cholesterol, together with its metabolites and precursors, regulates tumorigenesis and promotes biological process, such as oncogene-driving pathways, ferroptosis, and TME in malignancies [21]. In this respect, cholesterol inhibitors, including statins, are utilized in tumor therapies [22]. However, the role of cholesterogenesis in cancer remains arguable, and the efficacy of statins in regulating cancer has shown mixed effects. In addition, distinct responses exist due to tumor heterogeneity [23, 24]. However, the expression levels of mitochondrial pyruvate complex 1 (MPC1) and mitochondrial pyruvate complex 2 (MPC2) regulate malignancy outcomes [25], and this indicates the distinct performance of pyruvate flow in differentiated malignancy types. In this respect, it also shows that balanced glycolytic and cholesterogenic pathways jointly modulate tumor progression.

Based on previous studies of melanoma subtypes [5, 6], we aimed to define new subtypes from a metabolic perspective by using different metabolic levels. We used a large-patient cohort from TCGA (https://www.cancer.gov/about-nci/organization/ccg/research/structural-genomics/tcga) to explore novel subclassifications in melanoma based on glycolysis-cholesterogenesis differential expressed genes, and we then compared the characteristics of the discovered subtypes and further validated the subtyping efficacy and reproducibility. We determined that the subtype with the highest glycolysis and lowest cholesterol synthesis had the worst prognosis. We subsequently explored the nature of the glycolytic subgroup and finally developed a risk prognostic model based on the glycolytic subgroup to provide a quantitative method that involves an enhanced biological understanding and which can be used to develop clinical strategies for SKCM management.

## Results

### The identification of four metabolic subgroups along the glycolysis-cholesterol synthesis axis

**Fig. 1:**
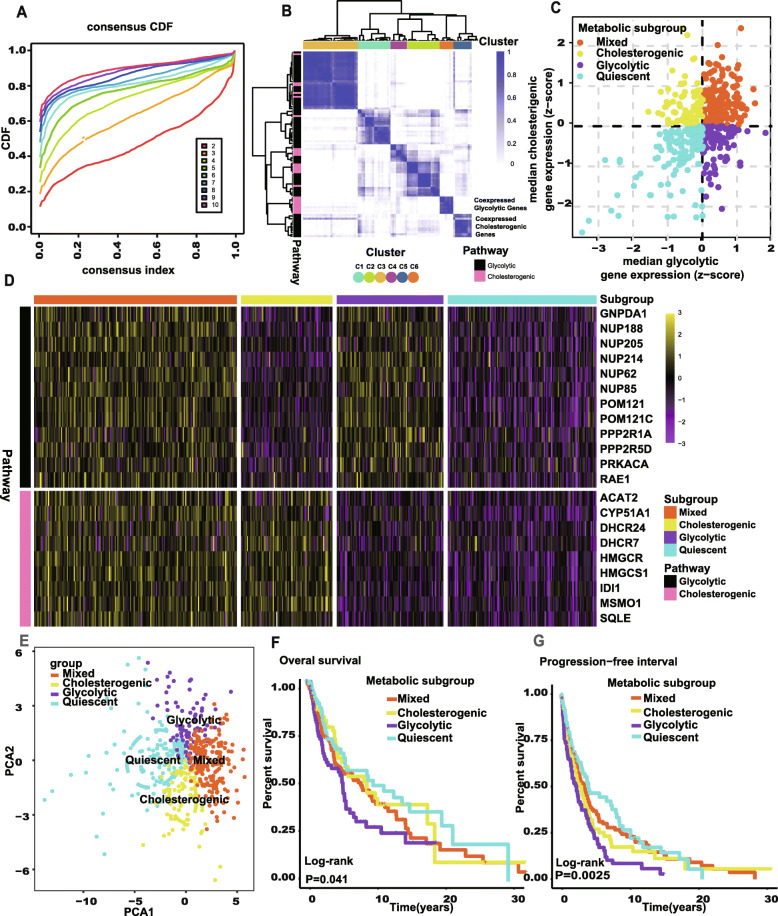
Stratification of SKCM tumors based on expression of glycolysis and cholesterogenic genes. **A** The CDF curve under different values of *k*. The value of k represents the number of clusters during the consensus cluster. When the optimal *k* value is reached, the area under the CDF curve will not significantly increase with the increase of k value. **B** Heatmap depicting consensus clustering solution (*k*=6) for glycolysis and cholesterogenic genes in SCKM samples (*n*=469). **C** Scatter plot showing median expression levels of co-expressed glycolytic (*x*-axis) and cholesterogenic (*y*-axis) genes in each SKCM sample. Metabolic subgroups were assigned on the basis of the relative expression levels of glycolytic and cholesterogenic genes. **D** Heatmap depicting expression levels of co-expressed glycolytic and cholesterogenic genes across each subgroup. **E** PCA showed that patients in the different subtgroups were significantly different from each other. **F** Kaplan-Meier survival curves for patients in the different subgroups. Log-rank test P values are shown. The clinical outcome endpoint is OS. **G** Kaplan-Meier survival curves for patients in the different subgroups. Log-rank test *P* value is shown. The clinical outcome endpoint is PFI. SKCM, skin cutaneous melanoma; CDF, cumulative distribution function; PCA, principal components analysis; OS, overall survival; PFI, progression-free interval. And *P* < 0.05 is defined as statistically significant

The gene expressions of 366 SKCM tumor samples obtained from the TCGA were used to screen the co-expressed glycolytic and cholesterogenic genes. Consensus clustering was conducted on 72 genes in the glycolytic pathway and 25 genes in the cholesterogenic pathway. According to the results, the best grouping scheme was obtained with 6 (*k* = 6) gene clusters (Fig. 1A), where the method used to determine the optimum *k* value is provided in the work of Wilkerson et al. [26]. Hierarchical clustering was then conducted based on the consensus matrix generated. As shown in Fig. 1B, genes in C5 (defined as glycolytic co-expressed genes) all belonged to the glycolytic pathway and genes in C6 (defined as cholesterogenic co-expressed genes) all belonged to the cholesterogenic pathway. The median expressions of these glycolytic and cholesterogenic co-expressed genes were then acquired from 366 SKCM tumor samples, and four metabolic subgroups were subsequently identified based on the median expressions (Fig. 1C). The expressed levels of these selected genes were visualized across four subgroups (Fig. 1D), and principal component analysis (PCA) was used to illustrate the difference between the four subgroups (Fig. 1E).

The results showed that patients in the glycolytic subgroup had the worst prognosis (Fig. 1F and G), which indicates that SKCM with higher glycolysis and lower cholesterol synthesis may have more aggressive characteristics. The clinically relevant information relating to the four subtypes is presented in Table 1. We found that the overall survival (OS) rates and progress-free intervals (PFIs) differed significantly among patients in the different four subtypes (log-rank test, *P* = 0.024 and < 0.001). There were no statistical differences between the other clinical characteristics of patients, but this result could relate to the large number of categories used with respect to certain characteristics.

**Table 1:** Correlations between the four metabolic subtypes and clinical characteristics in the TCGA cohort

	Mixed (*n*=123)	Cholesterogenic (*n*=55)	Glycolytic (*n*=76)	Quiescent (*n*=90)	Total (*n*=344)	*P* value
Age						
Mean (SD)	57.5 (15.4)	54.6 (17.1)	61.1 (14.4)	57.5 (16.5)	57.8 (15.8)	0.159^a^
Median [MIN,MAX]	57 [15,90]	56 [19,82]	63 [18,90]	57 [18,90]	58 [15,90]	
Gender						
Female	44 (35.8%)	18 (32.7%)	29 (38.2%)	38 (42.2%)	129 (37.5%)	0.668^b^
Male	79 (64.2%)	37 (67.3%)	47 (61.8%)	52 (57.8%)	215 (62.5%)	
Race						
Asian	5 (4.1%)		2 (2.6%)	2 (2.2%)	9 (2.6%)	
White	118 (95.9%)	55 (100.0%)	74 (97.4%)	88 (97.8%)	335 (97.4%)	
Pathological T						
T0	11 (8.9%)	4 (7.3%)	3 (3.9%)	5 (5.6%)	23 (6.7%)	0.4^b^
T1	14 (11.4%)	8 (14.5%)	6 (7.9%)	8 (8.9%)	36 (10.5%)	
T2	29 (23.6%)	11 (20.0%)	17 (22.4%)	15 (16.7%)	72 (20.9%)	
T3	27 (22.0%)	12 (21.8%)	25 (32.9%)	18 (20.0%)	82 (23.8%)	
T4	42 (34.1%)	20 (36.4%)	24 (31.6%)	44 (48.9%)	130 (37.8%)	
Tis			1 (1.3%)		1 (0.3%)	
Pathological N						
N0	77 (62.6%)	35 (63.6%)	39 (51.3%)	46 (51.1%)	197 (57.3%)	0.693^b^
N1	22 (17.9%)	10 (18.2%)	14 (18.4%)	18 (20.0%)	64 (18.6%)	
N2	13 (10.6%)	5 (9.1%)	11 (14.5%)	12 (13.3%)	41 (11.9%)	
N3	11 (8.9%)	5 (9.1%)	12 (15.8%)	14 (15.6%)	42 (12.2%)	
Pathological M						
M0	122 (99.2%)	51 (92.7%)	72 (94.7%)	87 (96.7%)	332 (96.5%)	0.077^b^
M1	1 (0.8%)	4 (7.3%)	4 (5.3%)	3 (3.3%)	12 (3.5%)	
Pathological stage						
Stage I	34 (27.6%)	10 (18.2%)	13 (17.1%)	13 (14.4%)	70 (20.3%)	0.143^b^
Stage II	39 (31.7%)	22 (40.0%)	24 (31.6%)	31 (34.4%)	116 (33.7%)	
Stage III	49 (39.8%)	18 (32.7%)	35 (46.1%)	41 (45.6%)	143 (41.6%)	
Stage IV	1 (0.8%)	4 (7.3%)	4 (5.3%)	3 (3.3%)	12 (3.5%)	
I/II NOS		1 (1.8%)		2 (2.2%)	3 (0.9%)	
Overall survival						
Survival	60 (48.8%)	26 (47.3%)	32 (42.1%)	58 (64.4%)	176 (51.2%)	0.024^c^ (*)
Death	63 (51.2%)	29 (52.7%)	44 (57.9%)	32 (35.6%)	168 (48.8%)	
Progress-free interval						
Free survival	37 (30.1%)	11 (20.0%)	16 (21.1%)	43 (47.8%)	107 (31.1%)	<0.001^c^ (*)
Death or progression	86 (69.9%)	44 (80.0%)	60 (78.9%)	47 (52.2%)	237 (68.9%)	

*T*, tumor; *N*, lymph node; *M*, metastasis; *P* values were calculated by the Kruskal test^a^, chi-square test^b^, or log-rank test^c^; *statistically significant

### Differences in somatic mutations across the metabolic subgroups

**Fig. 2:**
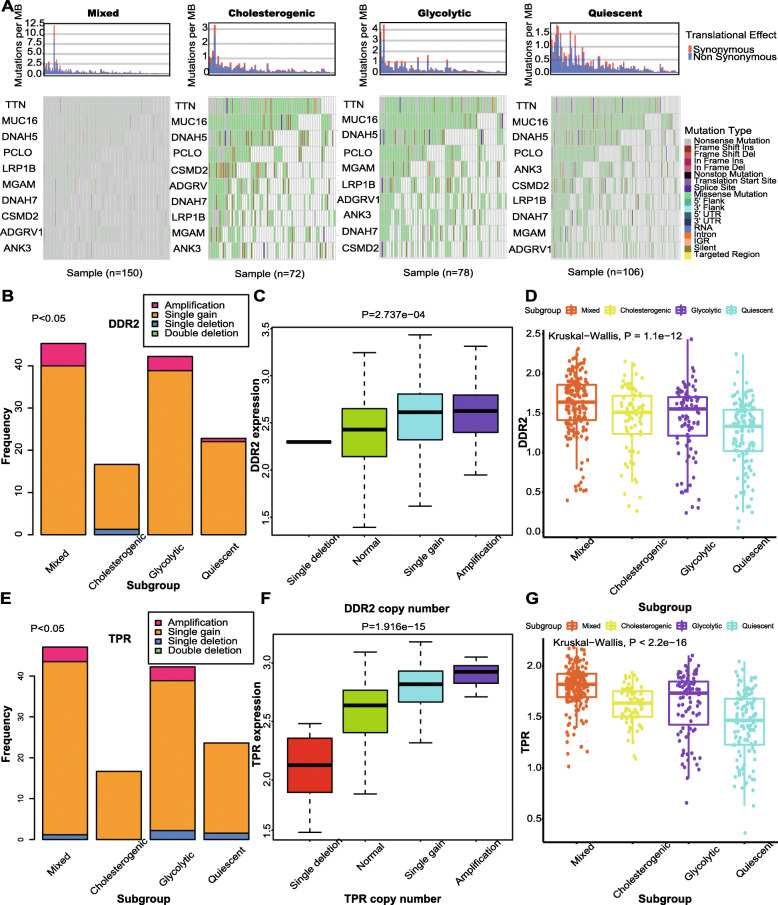
Mutational landscape across metabolic subgroups of SKCM. **A** The map of waterfall depicting the distribution of SNV events affecting frequently mutated genes in SKCM across the metabolic subgroups. **B** Bar plot illustrating the CNV of DDR2 across the metabolic subgroups. Chi-square test *P* value is shown. **C** Boxplot depicting the relationship of types of CNV and the expression of DDR2. **D** Boxplot depicting the expression levels of DDR2 across the metabolic subgroups. **E** Bar plot illustrating the CNV of TPR cross the metabolic subgroups. Chi-square test *P* value is shown. **F** Boxplot depicting the relationship of types of CNV and the expression of TPR. **D** Boxplot depicting the expression levels of TPR across the metabolic subgroups. SKCM, skin cutaneous melanoma; SNV, simple nucleotide variation; CNV, copy number variation. And *P* < 0.05 is defined as statistically significant

As shown Fig. 2A, we found that greater numbers of simple nucleotide variations (SNVs) of *MGAM* occurred in the glycolytic subgroup. *MGAM* is a member of the glycoside hydrolase family 31, and it is involved in galactose metabolism and metabolic pathways [27]. No studies to date have revealed a correlation between the SNVs of *MGAM* and the progress of the cancer, and we thus considered that to evaluate *MGAM* in cancer, glycolysis might be a good entry point. We therefore selected genes that had significantly different copy number variation (CNV) statuses across the metabolic subgroups. Of these, *DDR2* and *TPR* were filtered out for their high CNV frequencies in both the glycolytic and mixed subgroups. *DDR2* promotes the migration and invasion of metastatic melanoma cells [28], and *DDR2* inhibition makes tumors vulnerable to anti-PD-1 immunotherapy [29]. *TPR* can promote cell proliferation in colorectal cancer via binding with GSK3β [30]. In Fig. 2B and E, we found that amplifications or gains of *DDR2* or *TPR* were greater in the glycolytic and mixed subgroups (chi-square test *P* value < 0.05). Not unexpectedly, the gene levels of *DDR2* or *TPR* were found to be higher in the glycolytic or mixed subgroups, as shown in Fig. 2D and G (Kruskal-Wallis *P* value < 0.05). There is thus a relationship between these results and the worse prognoses of the glycolytic and mixed subgroups, and it is considered that when conducting research targeting *DDR2* or *TPR*, the activity of glycolysis should be studied.

### Positive correlation between tumor purity and metabolic status

**Fig. 3:**
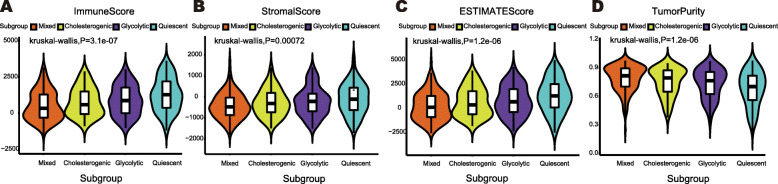
Evaluation of tumor purity across metabolic subgroups by ESTIMATE. **A** Violin plot illustrating the immune score across metabolic subgroups. **B** Violin plot illustrating the stromal score across metabolic subgroups. **C** Violin plot illustrating the ESTIMATE score across metabolic subgroups. **D** Violin plot illustrating the tumor purity across metabolic subgroups. Kruskal-wallis test *P* values are shown. And P < 0.05 is defined as statistically significant

According to the results of ESTIMATE, the immune score and stromal score were negatively correlated with the metabolic status, as shown in Fig. 3A and B (the mixed < the cholesterogenic < the glycolytic < the quiescent), and the result of the ESTIMATE score showing the sum of the immune and stromal scores is shown in Fig. 3C. The results indicate that there is a decrease in the number of immune cells and stromal cells infiltrated in the microenvironment of tumor tissue with an increase in the metabolic state. Tumor purity was then calculated using the ESTIMATE scores, and a higher metabolic status was found to be associated with higher tumor purity, as shown in Fig. 3D. This result suggests that a high metabolic status relates to high tumor tissue purity. In addition, tumors with high cholesterol synthesis were found to have a higher purity than tumors with high glycolysis (Fig. 3D).

### Glycolytic subgroup has a higher proliferation level

**Fig. 4:**
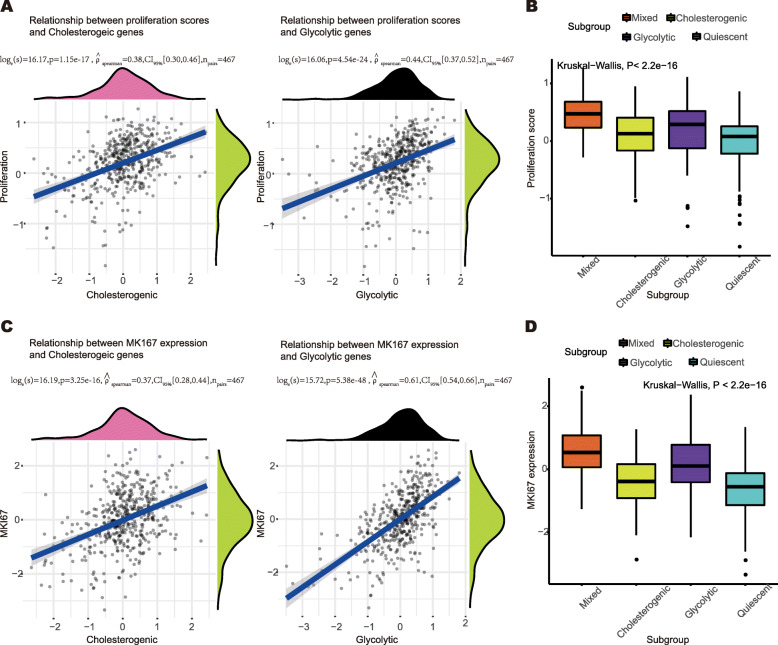
The proliferation status across metabolic subgroups. **A** Scatter plot depicting the correlation between the proliferation score and median expression of cholesterogenic genes (left) and glycolytic genes (right). The Spearman’s test *P* values are shown. **B** Boxplot illustrating proliferation score across metabolic subgroups. Kruskal-wallis test *P* value is shown. **C** Scatter plot depicting the correlation between the MKI67 expression and median expression of cholesterogenic genes (left) and glycolytic genes (right). The Spearman’s test *P* values are shown. **D** Boxplot illustrating the MKI67 expression across metabolic subgroups. Kruskal-wallis test *P* value is shown. *P* < 0.05 is defined as statistically significant

As shown in Fig. 4A, the proliferation score was positively correlated with the median expression of cholesterogenic genes (rho = 0.38, *P* < 0.05) and glycolytic genes (rho = 0.44, *P* < 0.05), and a higher proliferation score level was found in the glycolytic subgroup, as shown in Fig. 4B (Kruskal-Wallis *P* < 0.05). Similarly, the expression level of *MKI67* was positively correlated with the median expression of cholesterogenic genes (rho = 0.37, *P* < 0.05) and glycolytic genes (rho = 0.61, *P* < 0.05), as shown in Fig. 4C. Furthermore, the glycolytic group was found to have a higher expression level of *MKI67*, as shown in Fig. 4D (Kruskal-Wallis *P* < 0.05). These findings confirm that tumors in the glycolytic subgroups identified have greater proliferation abilities.

### Gene expressed networks and biological activities related to glycolysis and cholesterol synthesis

**Fig. 5:**
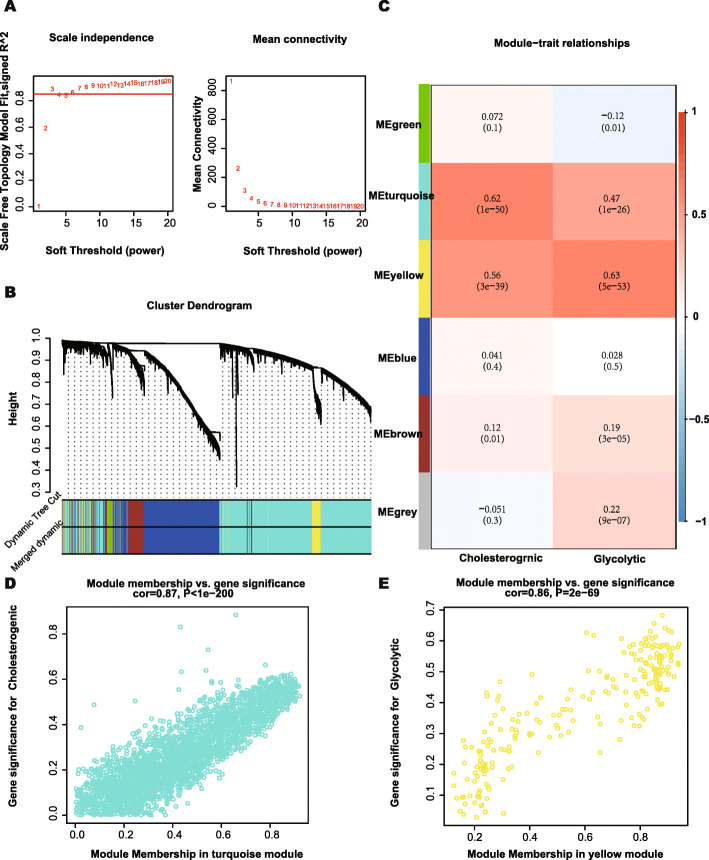
WGCNA to identify similar genes networks of cholesterogenic genes and glycolytic genes. **A** The relationship of soft threshold and TOM-based dissimilarity (left). The relationship of soft threshold and mean connectivity (right). **B** After the dynamic of cut and merged, a total of 6 gene modules were finally generated. **C** Heat map for the correlation of gene modules and phenotypes. **D** Scatter plot depicting the correlation between gene significance and module membership of genes in turquoise network. **E** Scatter plot depicting the correlation between gene significance and module membership of genes in yellow network. WGCNA, weighted correlation network analysis; TOM, topological overlap matrix. And *P* < 0.05 is defined as statistically significant

In this study, four metabolic subgroups were identified via different metabolic statuses, as represented by the median expression of glycolytic and cholesterogenic genes. WGCNA was then conducted to find more genes related to glycolysis and cholesterol syntheses and to conduct subsequent research. According to Fig. 5A, when the soft threshold was equal to 3, the gene networks satisfied both a high degree of internal connectivity and a high gene similarity. The gene networks with similarity were then merged and six networks represented by different colors were finally identified (Fig. 5B). The relationships between the gene networks and the median expressions of cholesterogenic and glycolytic genes were then explored, and the turquoise and yellow networks were found to be most related to cholesterol synthesis and glycolysis, respectively (Fig. 5C). The gene significance and module membership of genes in the turquoise and yellow networks are shown in Fig. 5D and E, and the values of these variables exhibit strong positive correlations. It is considered that gene significance could reflect the representativeness of a gene in the corresponding phenotype, and that membership could represent the correlation between a gene and its networks.

**Fig. 6:**
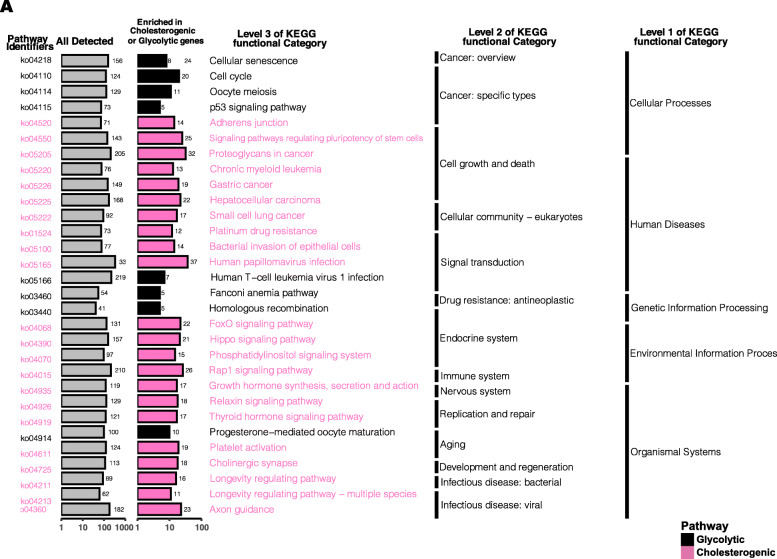
The results of KEGG analysis in cholesterogenic genes and glycolytic genes. KEGG, Kyoto Encyclopedia of Genes and Genomes. *P* < 0.05 is defined as statistically significant

The genes in the turquoise and yellow networks were used in a KEGG enrichment analysis (*P* < 0.05). As shown in Fig. 6A, glycolysis and cholesterol synthesis are related to cellular processes, human disease, and genetic information processing (level 1 of the KEGG functional category). Furthermore, the p53 signaling pathway, cell cycle, cellular sentence, and homologous recombination are enriched in glycolysis, whereas the adherence junction, platinum drug resistance, and proteoglycans in cancer are enriched in cholesterol synthesis.

### Development of a prognostic risk model

**Fig. 7:**
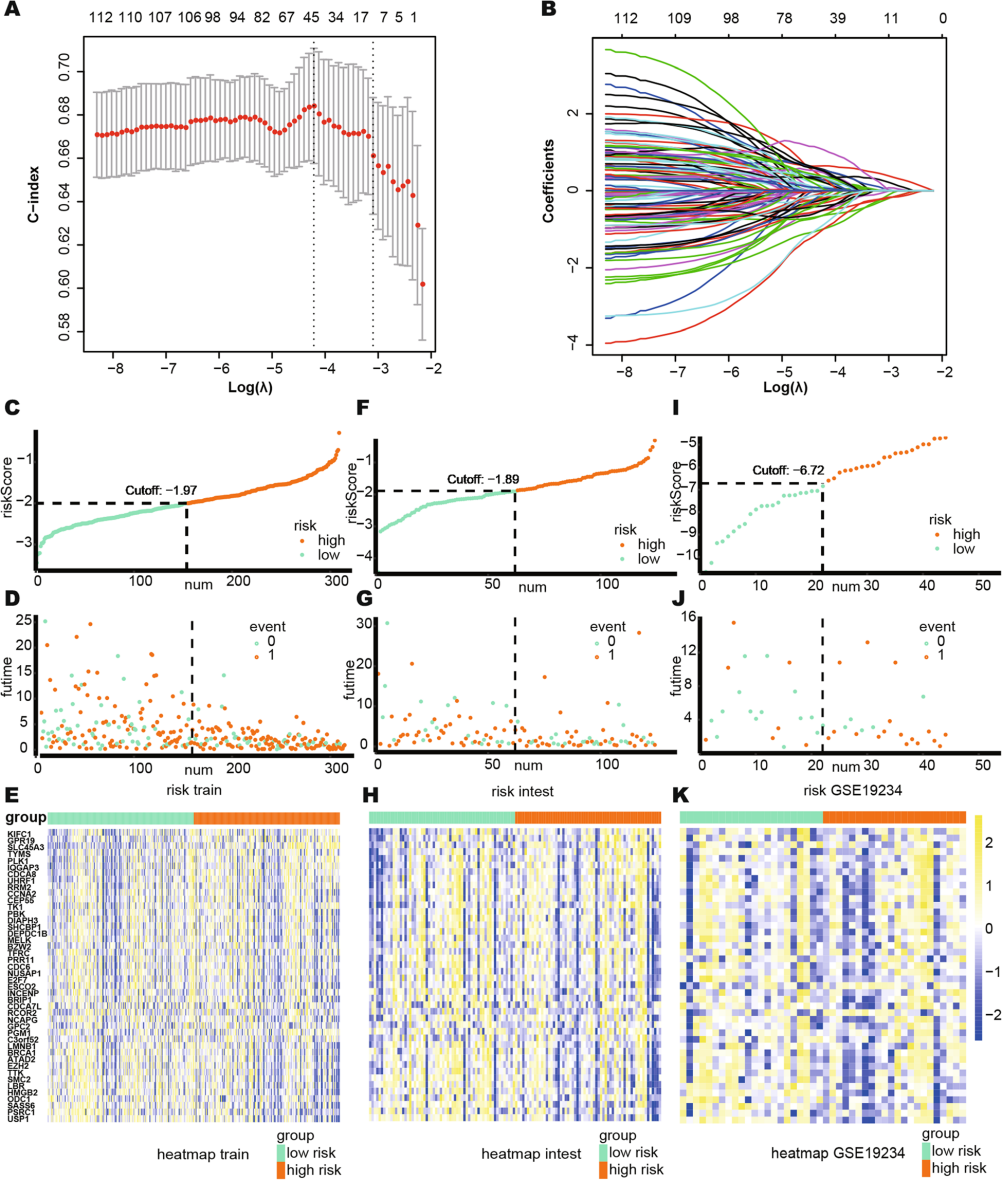
Build the risk model by LASSO. **A** Cross validation based on C-index to determine the best choice of genes in the model. **B** Genes in the different combinations and their corresponding coefficients. **C**–**E** Patients of training set were arranged in the same ascending order of the risk score. **F**–**H** Patients of internal validation set were arranged in the same ascending order of the risk score. **I**–**K** Patients of GSE19234 data set were arranged in the same ascending order of the risk score. **C**, **F**, **I** Patients were divided into different risk levels according to the median of the risk scores in their respective data sets. **D**, **G**, **J** The relationship between the survival outcome and risk levels of patients. Low-risk patients were shown on the left side of the dotted line and high-risk patients were shown on the right side. **E**, **H**, **K** Heat maps for the genes in the signature. LASSO, least absolute shrinkage and selection operator. And *P* < 0.05 is defined as statistically significant

Patients in the glycolytic subgroup were found to have the worst prognosis, and we thus used the genes in the yellow network, which are most related to glycolysis, to train the risk model. The C-index was set as the reference in the cross validation to select the optimum least absolute shrinkage and selection operator (Lasso) model for the training group. As shown in Fig. 7A, the 44-genes risk model had the highest C-index, and Fig. 7B shows the changes in the coefficients of different genes during the cross validation.

It was then possible to calculate the risk score of each patient to evaluate their risk level using the following equation,

$$ \mathrm{Risk}\ \mathrm{score}={\sum}_{n=1}^{44}\left({\mathrm{coefficient}}_n\times \mathrm{expression}\ {\mathrm{of}\ \mathrm{gene}}_n\right), $$

and the genes and their coefficients are shown in Table 2. Patients in the training group, internal validation group, and the GSE19234 data set were then ranked in an ascending order of risk score. Due to batch effect across different platforms, the median of the risk scores of each group was selected as the cut-off value to divide patients into high risk and low risk (Fig. 7C–H).

**Table 2:** Univariate Cox results and Lasso coefficients for risk score formula of genes

	HR	95% CI	*P* value	Lasso coefficient
KIFC1	1.7	(1.1–2.6)	0.016	0.345468464
GPR19	1.6	(1.1–2.4)	0.016	0.272311029
SLC45A3	1.6	(1.1–2.2)	0.016	0.645641342
TYMS	1.5	(0.98–2.2)	0.064	0.525818769
PLK1	1.5	(1–2.3)	0.029	0.111452595
IQGAP3	1.5	(1–2.3)	0.027	0.34671539
CDCA8	1.5	(0.98–2.2)	0.06	1.060191747
UHRF1	1.4	(1–2.1)	0.047	0.464561353
RRM2	1.3	(0.91–1.9)	0.15	0.208350724
CCNA2	1.3	(0.87–1.9)	0.21	0.218089206
CEP55	1.3	(0.85–1.8)	0.26	0.276731331
TK1	1.3	(0.89–2)	0.17	−0.870357919
PBK	1.2	(0.82–1.6)	0.39	0.04492278
DIAPH3	1.2	(0.86–1.8)	0.25	0.546604919
SHCBP1	1.2	(0.79–1.8)	0.41	0.462985398
DEPDC1B	1.2	(0.8–1.8)	0.35	0.501249454
MELK	1.2	(0.8–1.7)	0.42	−0.311001546
BZW2	1.2	(0.81–1.9)	0.32	0.068764877
TFRC	1.1	(0.83–1.6)	0.43	0.066927634
PRR11	1.1	(0.76–1.6)	0.59	−0.193881148
CDC6	1.1	(0.76–1.7)	0.51	−0.27245389
NUSAP1	1.1	(0.72–1.6)	0.68	−0.059609277
E2F7	1.1	(0.76–1.6)	0.58	−0.984225964
ESCO2	1.1	(0.68–1.8)	0.71	0.514700781
INCENP	1.1	(0.67–1.7)	0.74	−0.484422932
BRIP1	1.1	(0.7–1.8)	0.61	−0.123046978
CDCA7L	1	(0.84–1.3)	0.71	−0.016050038
RCOR2	1	(0.77–1.3)	0.97	−0.183697727
NCAPG	1	(0.72–1.4)	0.9	−0.041934418
GPC2	1	(0.73–1.5)	0.85	0.279342277
PGM1	1	(0.7–1.4)	0.99	0.048131126
C3orf52	1	(0.68–1.6)	0.88	0.372435672
LMNB1	0.99	(0.67–1.5)	0.94	−0.388549559
BRCA1	0.98	(0.62–1.5)	0.93	−0.041497529
ATAD2	0.95	(0.63–1.4)	0.79	0.323810656
EZH2	0.93	(0.61–1.4)	0.75	−0.289895316
TTK	0.92	(0.63–1.3)	0.66	–0.623252924
SMC2	0.82	(0.57–1.2)	0.29	−0.120862539
LBR	0.8	(0.56–1.1)	0.22	−0.486321723
HMGB2	0.79	(0.54–1.2)	0.23	−0.222782483
ODC1	0.78	(0.58–1.1)	0.12	−0.502894698
SASS6	0.76	(0.48–1.2)	0.22	0.033157158
PSRC1	0.7	(0.47–1)	0.08	−0.63407795
USP1	0.67	(0.47–0.95)	0.024	−0.981142492

Forty-four genes were selected by Lasso, and univariate Cox regression was performed on them. *HR*, hazard ratio; *CI*, confidence interval; *Lasso*, least absolute shrinkage and selection operator

**Fig. 8:**
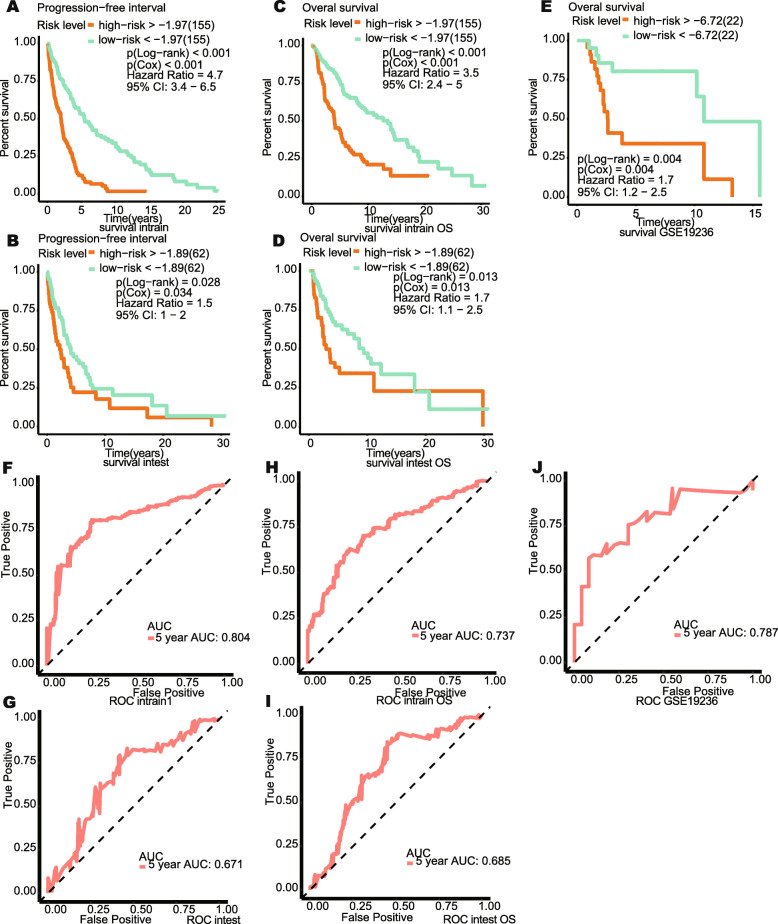
Verification of the effectiveness of the model. **A**–**E** Kaplan-Meier curve for survival analysis. **F**–**J** The ROC curve of 5-year follow-up time. **A**, **C**, **F**, **H** The results in the training set. **B**, **D**, **G**, **I** The results in the internal validation set. **E**, **J** The results in GSE19234. The clinical outcome endpoint in **A**, **B**, **E**, **F**, **G**, **J** was PFI. The clinical outcome endpoint in **C**, **D**, **H**, **I** was OS. AUC, area under curve; PFI, progression-free interval; OS, overall survival. And *P* < 0.05 is defined as statistically significant

**Fig. 9:**
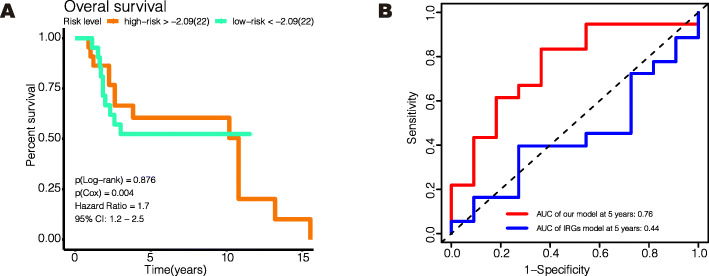
The comparison between our model and Liao’s immune-related model. **A** Kaplan-Meier curve for Liao’s immune-related model in GSE19234 (*n* = 44). The clinical outcome endpoint was OS. **B** The ROC curve of 5-year follow-up time for our model and Liao’s immune-related model in GSE19234 (*n* = 44). AUC, area under curve; OS, overall survival. And *P* < 0.05 is defined as statistically significant

The global expression levels of the 44 genes are shown in Fig. 7I–K. In the training group and internal validation groups, the high-risk patients had the worst prognosis for OS and PFI (Fig. 8A–D). In the GSE19234 group, the high-risk patients also had the worse prognosis for OS (Fig. 8E). In all these groups, the model showed a good predictive ability for OS or PFI (AUC > 0.65), as shown in Fig. 8F–I. Unlike our model, Liao’s immune-related model was unable to distinguish between high-risk and low-risk patients using an external dataset (GSE19234) (Fig. 9A, log-rank *P* = 0.876) [7], and their model provided a comparatively poorer performance in terms of calculating the AUC (Fig. 9B, 5 years AUC of Liao’s model = 0.44). To validate our model’s ability to obtain an independent prognostic factor, univariate and multivariate Cox regression analyses were conducted in the filtered international validation group (*n* = 92, patients with too many missing values of clinical information were removed) (Table 3), and we found that pathological T, pathological N, pathological M, and the risk model were able to independently predict a patient’s prognosis.

**Table 3:** Univariate and multivariate Cox regression for clinical variables and the prognostic risk model in international validation group

Prognostic index	Univariate analysis		Multivariate analysis	
	HR (95% CI)	*P* value	HR (95% CI)	*P* value
TCGA SKCM set (*n* = 92)				
Age	1.021 (1.003–1.039)	0.022	1.015 (0.995–1.035)	0.148
Gender	0.837 (0.496–1.411)	0.504		
Race	1.102 (0.15–8.089)	0.924		
Pathological stage	1.406 (1.023–1.932)	0.035	0.745 (0.43–1.292)	0.295
Pathological M	47.619 (10.351–219.066)	< 0.001	21.963 (4.23–114.032)	< 0.001
Pathological N	1.407 (1.081–1.831)	0.011	1.68 (1.114–2.533)	0.013
Pathological T	1.319 (1.1–1.582)	0.003	1.324 (1.058–1.658)	0.014
Risk score	1.699 (1.118–2.582)	0.013	1.351 (1.112–1.631)	0.027

*T*, tumor; *N,* lymph node; *HR*, hazard ratio; *CI*, confidence intervals

### Predicted use of drugs based on prognostic risk model

**Fig. 10:**
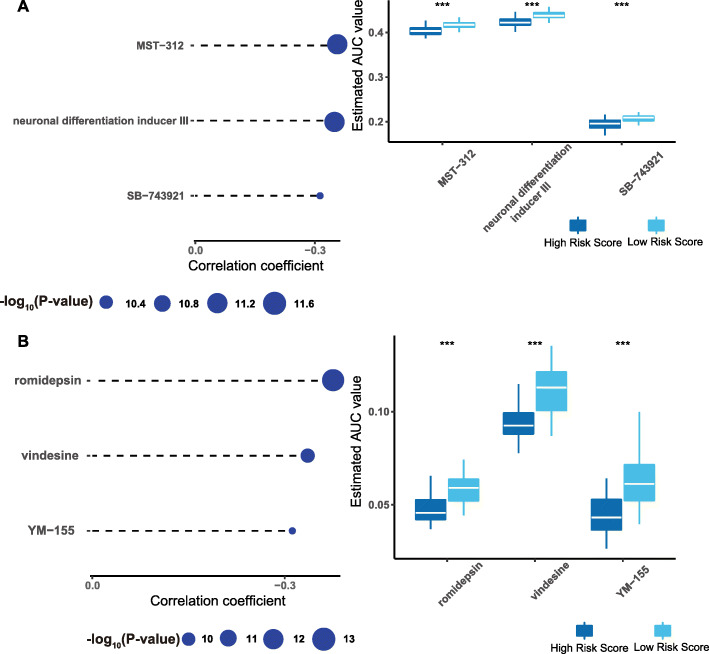
Identification of candidate agents with higher drug sensitivity in patients with high risk score. **A** The results of Spearman’s correlation analysis and differential drug response analysis of three CTRP-derived compounds. **B** The results of Spearman’s correlation analysis and differential drug response analysis of three PRISM-derived compounds. Note that lower values on the *y*-axis of boxplots imply greater drug sensitivity

We used compound data from CTRP and PRISM databases to predict which potential drugs could be administered to patients with high-risk scores. As shown in Fig. 10A, MST-312, neuronal differentiation inducer III, and SB-743921 showed high sensitivity for patients with high-risk scores, and Fig. 10B shows that romidepsin, vindesine, and YM-155 were highly sensitive for patients with high-risk scores.

## Discussion

In this research, we explored individual differences in SKCM from a metabolic perspective. Both glycolytic and cholesterogenic pathways orchestrate tumor metabolic adaptations and play roles in cancer progression and proliferation [31]. A previous study discussed the metabolic differential characteristics of pancreatic cancer, highlighted the existence of metabolic heterogeneity in tumors, and determined distinct profiles based on glycolytic and cholesterogenic genes [32]. We therefore hypothesized that SKCM could be subclassified based on glycolytic and cholesterol synthetic genes. A previous study identified TCGA SKCM subtypes based on genomic mutations [5], whereas our study focused on metabolic function to sub-classify SKCM. The results supplement those already published on the SKCM subtypes of TCGA.

We extracted glycolytic and cholesterogenic genes from “REACTOME_GLYCOLYSIS” (*n* = 72) and “REACTOME_CHOLESTEROL_BIOSYNTHESIS” (*n* = 25) gene sets, respectively, which were obtained from the Molecular Signatures Database v7.2 [33, 34]. To find the most representative genes and reduce noise, consistent clustering was used to reduce the number of genes. Ultimately six gene clusters were obtained (C1–6). C5 and C6 were then designated as the co-expressed cholesterogenic and glycolytic genes, respectively. Cluster C5 contained 9 cholesterogenic genes, including *ACAT2*, *CYP51A*, *DHCR24*, *DHCR7*, *HMGCR*, *HMGCS1*, *IDI1*, *MSMO1*, and *SQLE*, and Cluster C6 contained 12 glycolytic genes, including *GNPDA1*, *NUP188*, *NUP205*, *NUP214*, *NUP62*, *NUP85*, *POM121*, *POM121C*, *PPP2R1A*, *PPP2R5D*, *PRKACA*, and *RAE1*. Multiple genes have been digested in melanoma research and found to interfere with melanoma proliferation [35–43]. Based on above genes, the samples were divided to four subtypes: cholesterogenic, glycolytic, mixed, and quiescent groups.

Unexpectedly, the best prognosis was found in patients with the quiescent subtype, desert of both two metabolic pathway-related genes. The glycolytic subtype had the worst clinical outcomes and a significantly higher *MGAM* SNV rate than the others. *MGAM* (maltase-glucoamylase) belongs to the glycoside hydrolase family 31 and acts as a target in type II diabetes [44]. A higher expression of *MGAM* has been noted in luminal A breast cancers, and a meta-analysis identified that *MCAM* mutations are associated with a positive clinical benefit in non-small lung cancer cases [45, 46]. It is thus a promising focus for future glycolytic and cancer investigations. As mentioned in the results, the CNV statuses of *DDR2* and *TPR* were more significant in the glycolytic and mixed subtypes, which also support their association with a poor prognosis. It is of note that both higher glycolytic and cholesterogenic gene expressions indicated higher proliferation scores, although the slope of the glycolytic gene expressions was steeper.

Following subclassification and subsequent analyses, we succeeded in constructing four subtypes based on genes related to glycolysis and cholesterol synthesis pathways and ultimately recognized the glycolytic subtype as the group with the highest risk. We then tried to investigate further by analyzing the subclassifications, and we determined an optimum method for translating the results by constructing a risk predictive model.

The risk score modifier consisted of 44 genes relating to the glycolytic subtype, as it was found to have the worst prognosis. After conducting a literature review, we discovered that the roles of many genes in the classifier have been investigated with respect to melanoma and have been found to influence tumorigenesis. To be specific, *CDCA8* was positively related to the risk scores, and it was previously validated as being associated with tumor proliferations in melanoma by Chao et al. [47]. Similarly, *DEPDC1B SHCBP1*, *RRM2*, *PLK1*, and *UHRF1* have been found to promote melanoma proliferation and could be potential targets for treatment [48–52]; their coefficients were all positive, which implies that they had an additive effect on the risk scores.

The model was further validated using both internal and external validation processes. Potential drugs for high-risk patients were then investigated, and MST-312, neuronal differentiation inducer III, SB-743921, romidepsin, vindesine, and YM-155 were finally identified as high-sensitivity drugs for patients with high-risk scores. In this respect, MST-321 (telomerase inhibitor) induces apoptosis in multiple malignancy cells, such as myeloma cells and breast cancer cells [53, 54]. Neuronal Differentiation Inducer III is a neuronal differentiation inducing compound that can inhibit brain tumors through inducing brain stem cells differentiation [55], and SB-743921 (Molecular Formula: C31H33ClN2O3·HCl) acts as a potent and active KSP (kinesin spindle protein) inhibitor that inhibits multiple tumor growth and induces apoptosis [56, 57]. The efficacy of romidepsin, vindesine, and YM-155 in targeting melanoma has been previously validated [58–60]. Therefore, all these drugs could be considered for use in the clinical management of high-score SKCM patients.

With respect to its heterogeneity and plasticity, SKCM is a complicated disease and therapy intolerance and insensitivity may influence therapeutic efficacy. Our study provides a novel subclassification of SKCM and an associated risk score signature based on metabolic genes that could enhance biological understanding and clinical strategies.

However, although the results of this study are positive, certain limitations exist. First, although our research analyzed a large cohort of patients and verified the efficacy of the method using a dataset from another platform, a long-term prospective investigation is essential before applying the risk model in a clinical setting. Second, we used the median expression values for genes C5 and C6 with a cutoff of 0 to define subtypes with different glycolytic and cholesterogenic levels. However, the use of other clustering methods, such as K-means or Mclust, may be beneficial in the future. In addition, Lasso, smoothing clipped absolute deviation penalty (SCAD), and minimax concave penalty (MCP) regression methods are known to be the most effective for selecting variables. The objective function in Lasso is convex and easy to calculate, the coefficient of the compression independent variable is 0, and it has good robustness; therefore, it is especially practical. Several prognostic research studies have used Lasso when investigating tumor diseases [61–63]. Therefore, we used Lasso to develop our prognostic model in this study. However, the other two algorithms developed since Lasso, SCAD and MCP, are known to be occasionally better than Lasso when selecting important features [64]. Therefore, the failure in our study to use SCAD or MCP to screen the model is another limitation, and we will use these methods in future studies. Moreover, we determined several drugs that are highly sensitive in high-risk patients. Following a literature review, we found romidepsin, vindesine, and YM-155 have been shown to have antitumor effects in melanoma development, whereas investigations using MST-321, SB-743921 and Neuronal Differentiation Inducer III in SKCM are lacking. We therefore believe that they should be studied in the future.

## Conclusion

In this study, a dataset with 471 SKCM specimens was downloaded from The Cancer Genome Atlas (TCGA) database. We extracted and clustered genes from the Molecular Signatures Database v7.2 and acquired co-expressed glycolytic and cholesterogenic genes. We then subtyped the SKCM samples, and validated the efficacy of subtypes with respect to simple nucleotide variations (SNVs), copy number variation (CNV), patients’ survival statuses, tumor microenvironment, and proliferation scores. A survival analysis, SNV, CNV, and tumor environmental analyses were conducted, and the glycolytic subtype was found to have the worst prognosis in all the above aspects. We also explored the subtyping nature and built a 44-gene algorithm for predicting the SKCM prognosis in patients, and we validated the risk score signature in both internal and external independent cohorts. We further explored the sensitivity of using certain drugs in patients with different prognoses, and six drugs were finally identified to have higher sensitivities in high-risk SKCM patients (MST-321, SB-743921, Neuronal Differentiation Inducer III, romidepsin, vindesine, and YM-155).

## Methods and materials

### Data acquisition and processing

We downloaded datasets of SKCM samples from the TCGA database (https://portal.gdc.cancer.gov/) that included the following data: transcriptome profiling (RNA-seq, *n* = 471), simple nucleotide variations (SNVs), and copy number variations (CNVs). Normalization of the RNA-seq was chosen as the Fragments Per Kilobase Million (FPKM), and the FPKM format of the RNA-seq was transformed into a Transcripts Per Kilobase Million (TPM) format. Clinical information about SKCM patients was subsequently downloaded from (https://xenabrowser.net/datapages/), and survival data were obtained from an integrated TCGA pan-cancer Clinical Data Resource (CDR) [65]. According to the CDR, the clinical endpoints used for SKCM were selected as overall survival (OS) and progression-free interval (PFI). In addition, a dataset (GSE19234, *n* = 44) from the Gene Expression Omnibus (GEO, https://www.ncbi.nlm.nih.gov/geo/) with survival information was selected to validate the risk score model. In this study, we filtered out patients who had follow-up times of less than 30 days, and patients for whom survival information was missing were deleted.

### Identification of metabolic subgroups

First, we extracted glycolytic and cholesterogenic genes from the “REACTOME_GLYCOLYSIS” (*n* = 72) and “REACTOME_CHOLESTEROL_BIOSYNTHESIS” (*n* = 25) gene sets, respectively, where were obtained from the Molecular Signatures Database v7.2 [33, 34]. A consensus cluster was then conducted on these genes using the ConsensusClusterPlus R package [26]. The number of subsamples was 100, the proportion of items to samples was 0.8, the proportion of features to samples was 1, and the hierarchical linkage method for subsampling and the consensus matrix was Ward. D2. The consensus cluster results provided the co-expressed glycolytic and cholesterogenic genes. We also identified four metabolic subgroups based on the median expression of these two types of co-expressed genes and defined them as mixed (glycolytic median > 0, cholesterogenic median > 0), cholesterogenic (glycolytic median < 0, cholesterogenic median > 0), glycolytic (glycolytic median > 0, cholesterogenic median < 0), and quiescent (glycolytic median < 0, cholesterogenic median < 0) subgroups. A principal components analysis (PCA) was then conducted on all of the protein-coding genes to determine if patients in these four subgroups differed from each other. Finally, survival analyses were conducted based on the OS and PFI clinical endpoints, and the log-rank test P values were calculated.

### Analysis of SNV across metabolic subgroups

SNV data were obtained via the workflow of MuSE Variant Aggregation and Masking. We extracted the SNV matrix and divided it into four groups based on the metabolic subgroups. The genes were then ranked according to mutation frequency in descending order, and the top ten genes were selected to investigate their mutation statuses in the four subgroups via the GenVisR R package [66].

### CNV analysis across metabolic subgroups

First, we conducted the chi-square test on all genes with CNVs to select genes that had significantly different CNV statuses across the metabolic subgroups. Genes with expression levels that were not correlated with their CNV status were filtered out. Finally, genes with a high proportion of CNVs in the glycolytic or cholesterogenic subgroups were selected to reflect the characteristics of these subgroups.

### Analysis of tumor microenvironment across metabolic subgroups

Kosuke et al. developed an algorithm to determine tumor purity based on gene expression information [67] that can be used in the R package, ESTIMATE. The gene expressions of SKCM patients (*n* = 467) were input to ESTIMATE, and four scores were output. In this respect, the immune score reflected the abundance of immune cells around the tumor, the stromal score reflected the abundance of stromal cells around the tumor, and the ESTIMATE score was the sum of the immune and stromal scores, and it was negatively correlated with tumor purity.

### Analysis of the relationship between proliferation and metabolic subgroups

The proliferation score was acquired from the TCGA cohort research study [68]. The proliferative status was represented using the proliferation score and the expression of MKI67, which encodes KI67 and is the most common marker of cell proliferation. The correlation between the proliferative status and the median expression of glycolytic or cholesterogenic genes was explored using the Spearman’s test, and the ggstatsplot R package was used to visualize the results of Spearman’s rank correlation. The proliferative statuses across metabolic subgroups were visualized using the ggpubr R package, and the Kruskal-Wallis *P* values were calculated.

### Weighted gene co-expression network analysis (WGCNA)

All genes were arranged in descending order of variation between samples, and the top 5000 genes were selected for analysis using the R package, WGCNA [69]. According to the calculation conducted using this package, the soft threshold was set as 3. For gene module fusion, the cutoff value was set to 0.25. The results of WGCNA showed that gene modules were mostly correlated with the median expression of glycolytic and cholesterogenic genes, and this was thus selected for use in further research.

### Enrichment analysis of glycolytic and cholesterogenic phenotypes

As mentioned in the “Weighted gene co-expression network analysis (WGCNA)” section, we selected gene modules that were mostly correlated with the median expression of glycolytic and cholesterogenic genes. We then conducted an enrichment analysis on these genes via the clusterProfiler R package based on the gene sets within the Kyoto Encyclopedia of Genes and Genomes (KEGG) database [70, 71].

### Construction of a prognostic risk model via lasso

Genes in the yellow module were used to construct the risk model using Lasso, which is a popular machine learning method [72], and the glmnet R package was employed in this respect [73]. The parameters were set as follow: family = “cox”, type.measure = “C”, and parallel = TRUE, and the other parameters (not mentioned here) were set as default. Patients from the TCGA were divided into a training group (*n* = 310) and an internal validation group (*n* = 124) via the caret R package [74]. The model in the training group was generated by Lasso, and a survival analysis and time-dependent receiver operating characteristic curve (tdROC) were completed to validate the effectiveness of this model in the training group (*n* = 310), internal validation group (*n* = 124), and GSE19234 (*n* = 44). The proposed model was then compared to the existing one in GSE19234 (*n* = 44) in terms of the survival analysis and AUC using the Survival R package [7]. As a continuous risk score was obtained from the model, Cox test and log-rank test *P* values were simultaneously obtained. Since the risk scores were derived from training group, univariate and multivariate Cox regression analyses of the risk score and other clinical pathological variables were conducted only in the international validation group (*n* = 124). And due to some patients had too many missing values of clinical information, the patients in international validation group were filtered and 92 patients were screened out.

### Identification of candidate components with higher drug sensitivity in patients with high-risk score

The study of Paul Geeleher et al. demonstrated a method using only before-treatment baseline tumor gene expression data that could be used to predict the chemotherapeutic response of patients [75]. They tested a number of the plethora of common machine learning algorithms, including random forest, PAM, principal component regression, Lasso, ElasticNet regression, and ridge regression. Among these, ridge regression was consistently found to be the best performer, and it was noted to have the added advantage of being highly computationally efficient. In their method, the ridge regression tuning parameter was automatically selected, and to facilitate the use of their method, they developed an R package named pRRophetic [76]. With the help of pRRophetic R package, we predicted the sensitivity of SKCM patients from the TCGA to different agents using ridge regression based on data from CTRP2.0 and PRISM databases. Both datasets provided the area under the dose–response curve (AUC) values as a measure of drug sensitivity, and lower AUC values indicated an increased sensitivity to treatment. The components with significantly lower AUCs in high-risk patients were first selected, a Spearman’s correlation test between the AUC and the risk score was conducted, and components with significantly negative rho (rho < − 0.3) were retained. Results from the CTRP2.0 and PRISM databases are shown separately.

### Statistical analyses

All statistical analyses were conducted using R software 3.6.3. In all analyses, *p* < 0.05 was considered statistically significant. All of the R packages used in the various analytical procedures are listed in the associated corresponding chapters.

## Data Availability

The datasets analyzed during the current study are available in the TCGA Research Network [https://www.cancer.gov/tcga] and GEO [https://www.ncbi.nlm.nih.gov/geo/]. Annotated R code to reproduce all of the analysis in this study can be obtained by contacting the first author or the corresponding author.

## Abbreviations

SKCM: Skin cutaneous melanoma
TCGA: The Cancer Genome Atlas
SNV: Simple nucleotide variations
CNV: Copy number variation
TME: Tumor microenvironment
GLUT1: Glucose transporter isoform 1
HK1: Glycolysis-related genes hexokinase 1
LDH-A: Lactate dehydrogenase A
MCT1: Monocarboxylate transporters 1
MPC: Mitochondrial pyruvate complex
TCA cycle: Tricarboxylic acid cycle
